# Agrichemicals in surface water and birth defects in the United States

**DOI:** 10.1111/j.1651-2227.2008.01207.x

**Published:** 2009-04

**Authors:** Paul D Winchester, Jordan Huskins, Jun Ying

**Affiliations:** 1 Section of Neonatal-Perinatal Medicine, Indiana University School of MedicineIndianapolis, IN, USApaul.winchester@ssfhs.org; 2 Indiana University School of MedicineIndianapolis, IN, USA; 3 Institute for the Study of Health, University of CincinnatiCincinnati, OH, USA

**Keywords:** Atrazine, Birth defects, Nitrates, Pesticides

## Abstract

Objectives: To investigate if live births conceived in months when surface water agrichemicals are highest are at greater risk for birth defects.

Methods: Monthly concentrations during 1996–2002 of nitrates, atrazine and other pesticides were calculated using United States Geological Survey's National Water Quality Assessment data. Monthly United States birth defect rates were calculated for live births from 1996 to 2002 using United States Centers for Disease Control and Prevention natality data sets. Birth defect rates by month of last menstrual period (LMP) were then compared to pesticide/nitrate means using logistical regression models.

Results: Mean concentrations of agrichemicals were highest in April–July. Total birth defects, and eleven of 22 birth defect subcategories, were more likely to occur in live births with LMPs between April and July. A significant association was found between the season of elevated agrichemicals and birth defects.

Conclusion: Elevated concentrations of agrichemicals in surface water in April–July coincided with higher risk of birth defects in live births with LMPs April–July. While a causal link between agrichemicals and birth defects cannot be proven from this study an association might provide clues to common factors shared by both variables.

## INTRODUCTION

The leading cause of infant mortality in the United States is birth defects (1), accounting for 20.1% of all infant deaths. There is a growing body of evidence that agrichemical exposures may contribute to birth defects (2–8).

Large-scale longitudinal human studies evaluating pesticide/nitrate exposure at the time of conception have not yet been performed in the United States. Concentrations of nitrates and pesticides in stream water may be an indication of human exposure levels. The United States Geological Survey's (USGS) National Water-Quality Assessment (NAWQA) study provides the most comprehensive national-scale analysis of pesticide occurrence and concentrations in streams and ground water. In the NAWQA study, pesticide concentrations were measured in water samples from 186 stream sites representing 51 hydrological systems from 1991 to 2002. The NAWQA study units account for 70% of total water use and 50% of the United States drinking water. Pesticides were found to be present in most stream water samples and over half of the ground water samples. Seasonal patterns of pesticide concentrations were found with the highest monthly concentrations in May and June (8). The study also found that 90% of pesticide exposure is to mixtures versus individual pesticides.

The USGS indicated a strong relationship between pesticide occurrence in water samples and their use each year. Studies of pesticide occurrence in humans also correlated with pesticide applications and peaked in the spring months (9–11).

The present study relies on the general findings by USGS, the Environmental Protection Agency (EPA) and other agencies indicating that seasonal variations in nitrates, atrazine and other pesticides may serve as markers for annual agricultural and urban pest-control activities. In the present investigation we sought to answer a qualitative question; are annual peaks in pesticides and nitrates (typically from April to July) correlated with greater risk to pregnancies conceived in those months? If no increase in birth defects were found in April–July conceptions it might be inferred that the contaminant peaks pose little threat to human reproductive success.

## METHODS

Surface water nitrates, atrazine and all other measured pesticide concentrations (agrichemicals) were obtained monthly for each year between 1996 and 2002 from the USGS NAWQA database. Monthly pregnancy and birth outcome data were obtained from the Centers for Disease Control (CDC) natality database for the same years 1996–2002. Year of delivery, month of last menstrual period (LMP), presence of any birth defect and category of birth defect were recorded for each live birth. Maternal risk factors and demographics including alcohol use, tobacco use, diabetes, age, race and metropolitan or non-metropolitan residence were also recorded. Mother's month of LMP was used as a proxy for the time of conception and all birth defect rates were calculated based on cases per 100 000 live births for each LMP month. Stillbirths and abortion data were not used.

### Measures, predictors and factors

Primary measures of interest are (i) dichotomous variables of total birth defects and individual birth defects and (ii) numerical variables of concentrations of agrichemicals including atrazine, nitrate and other pesticides. The major factor of interest is the monthly or seasonal factor, that is the months April–July versus other months. Other predictors/factors include maternal risk factors, maternal demographics and year of birth.

### Statistical methods

We performed three major analyses in this study. First, dichotomous variables such as total and individual birth defects were assessed for their associations with the seasonal factor (a two-level factor of ‘peak’ season in months April–July and ‘off-peak’ season of other months) in a multivariate logistic regression model adjusting for other covariates such as maternal risk factors, maternal demographics and year. Second, agrichemicals were modelled with the seasonal factor using multivariate regression models, adjusting for years. Agrichemicals were log-transformed before performing multivariate regression analyses since their distributions were right skewed. Third, relationships between birth defects and agrichemicals were assessed using multiple logistic regression models, adjusting for maternal risk factors, maternal demographics and years. Both simple and multiple models were considered in this approach. The simple model used only one agrichemical as the major predictor of interest while the multiple models used all three agrichemicals as the predictor. All statistical analyses were performed using statistical software package SAS version 9.2 (Gary, NC). p-value <0.05 was considered statistically significant.

## RESULTS

### Baseline characteristics

A total of 30.11 million births were studied between 1996 and 2002. Table 1 shows women between ages 20 and 35 accounted for over 65% of total births, non-Hispanic whites over 59% and residents in rural areas less than 18%. One percent reported using alcohol during pregnancy, less than 13% using tobacco and less than 3% reported gestational diabetes.

**Table 1:** Summary of maternal demographics, maternal risk factors and overall birth defect rates by month of LMP (conception)

Variables		Total	Jan	Feb	Mar	Apr	May	Jun	Jul	Aug	Sep	Oct	Nov	Dec
Age*	<20	22.10	21.80	22.29	22.28	22.50	22.40	22.29	21.86	21.77	21.87	22.18	22.11	21.93
	20–35	65.87	65.79	65.55	65.58	65.52	65.67	65.84	66.15	66.24	66.25	65.88	65.88	66.04
	>35	12.03	12.41	12.17	12.14	11.98	11.93	11.86	12.00	11.98	11.88	11.95	12.01	12.03
Race/ethnicity*	White	59.93	60.23	58.25	58.73	59.64	60.59	61.31	62.47	62.07	61.33	60.50	59.71	59.78
	Black	14.88	14.38	15.38	15.46	15.46	14.98	14.60	13.83	13.89	14.22	14.73	14.86	14.59
	Other	5.54	5.60	5.64	5.65	5.44	5.41	5.40	5.43	5.41	5.36	5.30	5.34	5.41
	Hispanic	19.65	19.79	20.73	20.16	19.46	19.02	18.69	18.27	18.63	19.09	19.47	20.09	20.22
Metropolitan residence*		82.03	81.88	81.90	81.70	81.79	81.78	81.91	81.94	82.03	81.77	81.75	81.72	81.84
Alcohol*		1.00	0.95	0.98	0.97	1.06	1.05	1.03	1.00	0.99	1.00	1.01	0.99	0.98
Smoking*		12.40	12.29	12.62	12.56	12.80	12.47	12.31	11.98	11.97	12.33	12.62	12.51	12.31
Diabetes*		2.87	3.03	3.02	2.94	2.82	2.78	2.79	2.78	2.80	2.81	2.83	2.92	2.97
Total birth defects†		1588.52	1576.92	1567.17	1568.10	1612.86	1623.37	1636.19	1607.63	1584.55	1590.48	1566.56	1581.27	1547.09

* Values in cells are frequency (percentages) adjusted for years from 1996 to 2002.

† Values in cells are frequency (per 100 000 live births) of birth defects corresponding to mother's LMP months. Total number of live births (N) = 30.11 million.

### Birth defects

**Figure 1:**
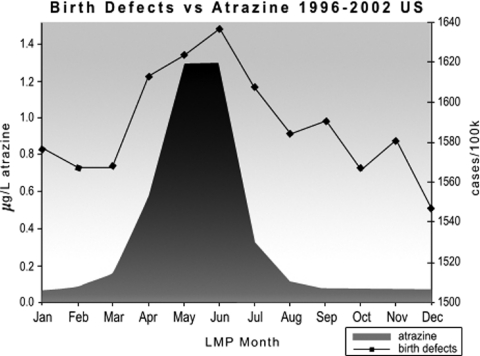
The United States birth defect rates by month of LMP versus atrazine concentrations.

**Figure 2:**
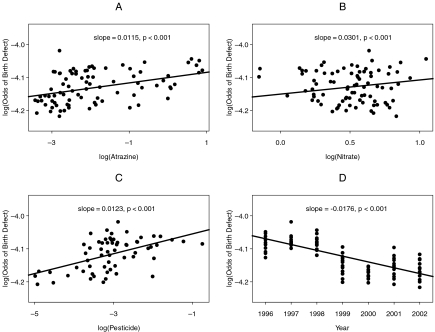
Plots of log odds of overall birth defect versus surface water concentrations (in log value) and year. Points in each plot represent all monthly observations in years 1996–2002. Solid lines and slopes were estimated from univariate logistic regression models. Units of atrazine, nitrate and pesticide concentrations are μg/L, mg/L and μg/L respectively.

Our study included 22 birth defect categories with the overall birth defect rate defined as any one birth defect. Table 1 and Figure 1 show the mean birth defect rates for each maternal LMP month. Birth defect rates were higher when mother's LMP was April–July. Table 2 shows that birth defect rates for April–July LMPs were significantly higher than birth defect rates for other LMP months (1621/100 000 vs. 1573/100 000 live births p < 0.01). Birth defects were positively associated to the maternal risk factors. Higher birth defects were found among mothers who had alcohol, smoking or diabetes. Nevertheless, mothers who didn't report having alcohol, tobacco or diabetes still had higher overall birth defect rates with LMPs in April–July than in other months. Figure 2D shows that birth defect rates decreased over the years of the study period. When individual birth defects were considered, spina bifida, circulatory/respiratory anomalies, tracheo-esophogeal defects, gastrointestinal defects, urogenital defects, cleft lip, adactyly, clubfoot, musculoskeletal anomalies, Down's syndrome and other birth defects were found to be significantly higher in April–July than in other months of the year (Table 3).

**Table 2:** Birth defects and surface water concentrations during April–July and other months

		Birth defect rate (per 100 000 live births)*		
		
Maternal risk factors		April–July	Other months	p-value†
All mothers:		1620.66 (34.09)	1572.58 (23.64)	<0.01
Alcohol	Yes	2591.95 (57.56)‡	2534.75 (40.52)‡	NS
	No	1465.80 (4.04)	1425.49 (2.81)	<0.01
Smoking	Yes	2111.56 (15.30)‡	2074.07 (10.56)‡	<0.05
	No	1371.57 (4.19)	1330.66 (2.89)	<0.01
Diabetes	Yes	2502.35 (31.96)‡	2472.75 (21.70)‡	NS
	No	1541.47 (4.25)	1492.62 (2.94)	<0.01
**Agrichemical**		**Surface water concentrations**§		
Atrazine (μg/L)		1.31 (0.20)	0.16 (0.02)	<0.01
Nitrate (mg/L)		1.94 (0.10)	1.65 (0.04)	<0.05
Pesticides (μg/L)		0.14 (0.05)	0.05 (0.01)	<0.01

* Values in cells are mean (SE) of birth defect rate (per 100 000 live births), estimated from multivariate logistical regression models adjusting for maternal demographics and year.

† p-values <0.05 indicate significant difference between months April–July and other months. NS = not significant with p > 0.05.

‡ The birth defect rate of the category ‘Yes’ is higher than that of the category ‘No’ with a p-value <0.01.

§ Values in cells are geometric mean (SE) of surface water concentrations, estimated from multivariate regression models adjusting for year.

**Table 3:** Individual birth defects by month of LMP (time of conception)

	Birth defects (per 100 000)*		
	
Birth defect type	April–July	Other months	p-value
Spina	21.93 (0.50)	20.31 (0.34)	<0.01
Circul	134.99 (1.25)	131.09 (0.85)	<0.01
Cleftlip	83.09 (0.98)	79.07 (0.66)	<0.01
Adactyly	85.65 (1.00)	81.88 (0.68)	<0.01
Musculo	223.49 (1.61)	217.36 (1.11)	<0.01
Down	46.23 (0.74)	43.22 (0.49)	<0.01
Othercon	455.89 (2.33)	443.89 (1.59)	<0.01
Tracheo	13.33 (0.39)	12.32 (0.26)	<0.05
Gastro	32.22 (0.61)	30.82 (0.42)	<0.05
Urogen	105.37 (1.11)	102.54 (0.76)	<0.05
Clubfoot	58.17 (0.82)	56.23 (0.56)	<0.05
Anen	10.70 (0.35)	10.67 (0.24)	NS^†^
Hydro	23.24 (0.52)	22.80 (0.36)	NS^†^
Micro	6.30 (0.27)	6.08 (0.18)	NS^†^
Nervous	20.97 (0.49)	20.98 (0.34)	NS^†^
Heart	118.85 (1.18)	117.44 (0.82)	NS^†^
Rectal	9.05 (0.32)	8.39 (0.22)	NS^†^
Omphalo	28.89 (0.58)	28.10 (0.40)	NS^†^
Genital	79.36 (0.96)	79.09 (0.66)	NS^†^
Renalage	13.99 (0.40)	13.52 (0.28)	NS^†^
Hernia	12.11 (0.38)	11.83 (0.26)	NS^†^
Chromo	36.24 (0.65)	34.91 (0.44)	NS^†^

* Values in cells are mean (standard error) adjusting for years.

† NS = not significant with p > 0.05. p-values are obtained from logistic regression models.

### Surface water concentrations

Table 2 shows atrazine, nitrate and other pesticides are the highest in April–July. The geometric means (standard errors) of atrazine, nitrate and other pesticides in April–June were 1.31 (0.20) μg/L, 1.94 (0.10) mg/L, and 0.14 (0.05) μg/L respectively; and were 8.2, 1.2 and 2.8 folds of those in other months (all p-values <0.05).

### Association between birth defects and surface water concentrations

Figure 2A–C) demonstrates that log odds of Birth Defects Are Positively Correlated With Atrazine, nitrate and other pesticide concentration levels in the simple models. Similar results were also found in the multiple analysis when all three geometrical predictors were included in the logistical regression model (p-values <0.05 for all three predictors). Table 4 demonstrates the effect of atrazine, nitrate and other pesticides. In general, individual birth defects rarely reached significance with individual contaminants. Only ‘other congenital anomalies’ reached significance in simple and multiple models with all three contaminant classes. In simple regression models, however, atrazine exposure increased the odds of 9 of 11 birth defects found to be associated with LMPs in April–July. This table contrasts with Table 4 in which all contaminants were significantly associated with any birth defects combined.

**Table 4:** Odds ratio (OR) of selected individual birth defects in relation to environmental contaminants (agrichemicals)

	Simple model*			Multiple model^†^		
		
Dependent variables	Atrazine (μg/L in log)	Nitrate (mg/L in log)	Other Pesticides (μg/L in log)	Atrazine (μg/L in log)	Nitrate (mg/L in log)	Other Pesticides (μg/L in log)
Spina	1.023 (1.000, 1.047)^‡^	1.016 (0.903, 1.143)	0.988 (0.949, 1.029)	1.018 (0.988, 1.050)	1.012 (0.883, 1.160)	0.973 (0.928, 1.020)
Circul	1.004 (0.995, 1.013)	1.068 (0.893, 1.151)	1.006 (0.990, 1.023)	1.006 (0.994, 1.018)	0.932 (0.882, 0.986)	1.007 (0.988, 1.027)
Tracheo	1.030 (1.001, 1.061)^‡^	0.959 (0.825, 1.115)	1.069 (0.986, 1.113)^§^	1.016 (0.978, 1.056)	0.941 (0.790, 1.122)	1.060 (1.001, 1.094)^‡^
Gastro	1.021 (1.003, 1.041)^‡^	0.974 (0.884, 1.074)	0.985 (0.951, 1.019)	1.024 (0.999, 1.051)^‡^	0.926 (0.825, 1.040)	0.972 (0.933, 1.012)
Urogen	1.007 (0.997, 1.017)	0.735 (0.613, 1.015)	1.021 (1.004, 1.040)^‡^	1.007 (0.994, 1.021)	0.982 (0.923, 1.044)	1.018 (0.957, 1.038)
Cleftlp	1.021 (1.009, 1.033)^§^	0.991 (0.933, 1.053)	0.999 (0.978, 1.020)	1.024 (1.009, 1.040)^§^	0.960 (0.895, 1.031)	0.983 (0.959, 1.008)
Adactyly	1.022 (1.011, 1.034)^§^	1.024 (0.965, 1.087)	1.023 (1.003, 1.045)^‡^	1.023 (1.007, 1.039)^§^	0.971 (0.906, 1.042)	1.008 (0.984, 1.032)
Clubfoot	1.016 (0.996, 1.028)^§^	0.993 (0.924, 1.067)	1.005 (0.980, 1.031)	1.014 (0.995, 1.033)	0.983 (0.903, 1.071)	0.996 (0.967, 1.025)
Musculo	1.015 (1.008, 1.022)^§^	1.025 (0.988, 1.064)	1.031 (1.018, 1.045)^§^	1.008 (0.999, 1.018)	1.004 (0.961, 1.049)	1.024 (1.009, 1.040)^‡^
Down	1.021 (1.005, 1.037)^§^	1.009 (0.930, 1.096)	0.999 (0.971, 1.028)	1.027 (1.005, 1.049)^‡^	0.982 (0.891, 1.082)	0.980 (0.947, 1.013)
Othercon	1.010 (1.005, 1.015)^§^	1.149 (1.120, 1.178)^§^	1.031 (1.022, 1.040)^§^	1.011 (1.002, 1.025)^§^	1.177 (1.143, 1.212)^§^	1.027 (1.016, 1.037)^§^

* Values in cells are mean (95% confidence interval) of odds ratio (OR) in response to one unit increase of each agrichemical predictor (in log). The ‘simple’ logistic regression models use only one agrichemical predictor and are adjusted for maternal risk factors, maternal demographics and years.

† Values in cells are mean (95% confidence interval) of odds ratio (OR) in response to one unit increase of each agrichemical predictor (in log). The ‘multiple’ logistic regression models use all three agrichemical predictors and are adjusted for maternal risk factors, maternal demographics and years.

‡ Indicates p < 0.05.

§ Indicates p < 0.01.

## DISCUSSION

This report has shown that during the period from 1996 to 2002 women in the United States with LMPs in April–July (i.e. the time of conception) were significantly more likely to have a live birth with a birth defect than in other months. The report further demonstrates, using NAWQA surface water samples that concentrations of atrazine, nitrates and other pesticides also were higher in the months of April–July. The correlation between birth defects, pesticides and nitrates was statistically significant.

Pesticides and nitrates, separately and in combination, have been linked to embryo toxicity and to untoward outcomes of pregnancy (12,13). Women's pesticide exposures through household gardening, professional application or living in close proximity to agricultural crops were associated with increased risks of offspring having neural tube defects and limb anomalies (14). Garry et al. found that in western Minnesota the rate of specific birth defects was elevated in pesticide applicators as well as the general population of western Minnesotans and that this risk was most pronounced for infants conceived in the spring (15). Specific birth defect categories showing significant increased risk in Garry's study were circulatory/respiratory, urogenital and musculoskeletal/integumental which are similar to the categories found in our study. Schreinemachers et al. found that infants in four wheat-producing states conceived in April–June, the time of herbicide application, were more likely to have circulatory/respiratory (excluding heart) malformations compared with births conceived during other months. She also found that counties with high wheat acreage had higher rates of heart malformations, musculoskeletal/integumental anomalies and infant death from congenital anomalies in males (16).

In Missouri men, high urine levels of atrazine, alachlor and diazinon were associated with abnormal sperm (9). The same study found that spring or summer samples were more likely to be abnormal than winter samples and that exposed men were frequently exposed to more than one pesticide causing many of the pesticide metabolites to be correlated. Thus, paternal as well as maternal exposures to pesticides might potentiate birth defects.

A causal link between birth defects and environmental nitrates/pesticides is plausible but not proven from this present ecological study. Nevertheless a statistically significant increased risk was found for any birth defect and for spina bifida, circulatory, tracheal, gastrointestinal, urogenital, musculoskeletal anomalies, cleft lip, adactyly, clubfoot and Down's syndrome in women with LMPs between April and July in the United Sates (Table 3). This period of increased risk is an important reproductive demographic.

Nitrates and pesticides occur as mixtures in most water samples (17). Recent observations in frogs, rats and other animals have demonstrated that individual chemicals at environmentally relevant concentrations may show little or no toxicity but when added together the effects are significantly more toxic or disruptive of vital endocrine functions (12,18,19). It is likely that other contaminants not specifically measured by the NAWQA study could also peak in April–July including air pollutants (20). Thus, the period of increased risk might not be associated solely with pesticides and nitrates.

This study has many limitations. Vital records have limited reliability and validity and should be used with caution (21). As of May 2001, 13 states plus the District of Columbia had only passive birth defects surveillance programmes (22).

Using NAWQA water data as a proxy for human exposure have significant limitations as well. Drinking water sources vary from surface to ground water and varied mixtures at different times of the year are common. Mean levels of nitrates and pesticides in NAWQA test sites are significantly higher than drinking water means would be in most locations. Nevertheless, in an EPA drinking water data sample from the same time period, peak frequency of pesticide detections were found in June, and correlated qualitatively with NAWQA surface water data. Atrazine was found in 57.9% of drinking water samples in Maryland (23) and 87% of drinking water samples in a sample of 12 Corn Belt states (17). A Canadian study of the northern Great Plains reported pesticides in numerous drinking water reservoirs, and depending upon location despite water treatment, 3 to 15 herbicides remained in drinking water (24).

Several studies in children and pregnant women using urine, amnionic fluid and meconium have demonstrated that from 89% to 100% of fetuses in the United States are exposed to pesticide *in utero* and most are exposed to mixtures of several pesticides (25). The importance of interactions between genetic susceptibility and *in utero* pesticide exposure has also been reported (26).

The National Health and Nutrition Examination Survey (NHANES) found that 95% of the United States population has measurable pesticide metabolites in urine samples (27). Although the atrazine mercapturate (AM) metabolite of atrazine was found in <5% of NHANES participants, Barr et al. have recently found that population-based atrazine exposures have been significantly underestimated for samples collected in the 1990's (28). The National Human Exposure Assessment Study in Maryland (NHEXAS-MD) demonstrated that over 80% of sampled individuals had at least one of three pesticide metabolites in their urine. The study found atrazine peaks occur in late summer and fall in the Baltimore area whereas the Midwest peaks occur in May and June (29).

## CONCLUSIONS

Birth defect rates in the Unites States were found to be highest for women conceiving in the spring and summer (maternal LMPs in April–July). This increase was significant for 11 of the 22 categories of birth defects reported in the CDC natality database from 1996 to 2002. A significant association was found between the months of increased risk of a birth defect (April–July) and increased levels of nitrates, atrazine and other pesticides in surface water. Critical time periods before and after conception may link seasonal peaks in environmental contaminants to certain birth defects.

## Abbreviations

LMP: first day of last menstrual period
NAWQA: National Water Quality Assessment Programme
USGS: The United States Geological Survey
CDC: Centers for Disease Control
OR: odds ratio
mg/L: milligrams per litre
μg/L: micrograms per litre
Anen: anencephalus
Spina: spina bifida/meningocele
Hydro: hydrocephalus
Micro: microcephalus
Nervous: other central nervous system anomalies
Heart: heart malformations
Circul: other circulatory/respiratory anomalies
Rectal: rectal atresia/stenosis
Omphalo: omphalocele/gastroschisis
Gastro: other gastrointestinal anomalies
Genital: malformed genitalia
Renalage: renal agenesis
Urogen: other urogenital anomalies
Cleftlp: cleft lip/palate
Adactyly polydactyly: syndactyly, adactyly
Clubfoot: club foot
Hernia: diaphragmatic hernia
Downs: Down syndrome
Chromo: other chromosomal anomalies
Musco: musculoskeletal
Othercon: other congenital anomalies
Tracheo: tracheoesophageal fistula
NHANES: National Health and Nutrition Examination Survey
NHEXAS: National Human Exposure Assessment Study
AM: atrazine mercapturate
